# The Candidate Gene Approach

**Published:** 2000

**Authors:** Jennifer M. Kwon, Alison M. Goate

**Affiliations:** Jennifer M. Kwon, M.D., is an assistant professor of neurology and Alison M. Goate, D.Phil., is a professor of genetics in psychiatry at Washington University School of Medicine, St. Louis, Missouri. Both authors are researchers in the Collaborative Study of the Genetics of Alcoholism

**Keywords:** genetic theory of AODU (AOD [alcohol or other drug] use, abuse, and dependence), genetic linkage, genetic polymorphism, nucleotides, apolipoproteins, quantitative trait locus, alcohol dehydrogenases, aldehyde dehydrogenases, Alzheimer’s disease

## Abstract

Alcoholism has a significant genetic basis, and identifying genes that confer a susceptibility to alcoholism will aid clinicians in preventing and effectively treating the disease. One commonly used technique to identify genetic risk factors for complex disorders such as alcoholism is the candidate gene approach, which directly tests the effects of genetic variants of a potentially contributing gene in an association study. These studies, which may include members of an affected family or unrelated cases and controls, can be performed relatively quickly and inexpensively and may allow identification of genes with small effects. However, the candidate gene approach is limited by how much is known of the biology of the disease being investigated. As researchers identify potential candidate genes using animal studies or linking them to DNA regions implicated through other analyses, the candidate gene approach will continue to be commonly used.

Family, twin, and adoption studies have indicated that alcoholism has a strong genetic component (Reich et al. 1999). Although researchers are still investigating its exact nature, several genes of varying effect that confer a susceptibility to alcoholism (i.e., susceptibility genes) likely play a role. Identification of these genes will aid researchers and clinicians in preventing and effectively treating this disorder.

The search for alcoholism susceptibility genes centers on two major techniques, linkage mapping and the candidate gene approach. Linkage mapping, also called positional cloning, is the process of systematically scanning the entire DNA contents (i.e., the genomes) of various members of families affected by the disorder using regularly spaced, highly variable (i.e., polymorphic) DNA segments whose exact position is known (i.e., genetic markers). Using those families, investigators can identify genetic regions associated or “in linkage” with the disease by observing that affected family members share certain marker variants (i.e., alleles) located in those regions more frequently than would be expected by chance. These regions can then be isolated, or cloned, for further analysis and characterization of the responsible genes. Linkage mapping techniques have already resulted in the identification of several potential DNA regions that may contain susceptibility genes for alcoholism (Reich et al. 1999). (For a review of the analogous approach in mice, see the article in this issue on quantitative trait locus [QTL] analysis by Grisel, pp. 169–174.) The primary advantage of linkage mapping is that investigators need no prior knowledge of the physiology or biology underlying the disorder being studied, which is important for complex disorders, such as alcoholism.

Whereas the linkage mapping approach is an unbiased search of the entire genome without any preconceptions about the role of a certain gene, the candidate gene approach allows researchers to investigate the validity of an “educated guess” about the genetic basis of a disorder. This approach involves assessing the association between a particular allele (or set of alleles) of a gene that may be involved in the disease (i.e., a candidate gene) and the disease itself. In other words, this type of association study tries to answer the question, “Is one allele of a candidate gene more frequently seen in subjects with the disease than in subjects without the disease?” The major difficulty with this approach is that in order to choose a potential candidate gene, researchers must already have an understanding of the mechanisms underlying the disease (i.e., disease pathophysiology). In contrast with linkage mapping studies, however, studies of candidate genes do not require large families with both affected and unaffected members, but can be performed with unrelated cases and control subjects or with small families (e.g., a proband and parents). Furthermore, candidate gene studies are better suited for detecting genes underlying common and more complex diseases where the risk associated with any given candidate gene is relatively small (Collins et al. 1997; Risch and Merikangas 1996). This article describes the methods used in candidate gene studies, including associated methodological and technical considerations, and reviews examples of this approach that are both related and unrelated to alcoholism. Because many of the best known examples of this approach have been conducted in humans, these studies have been highlighted. However, the overall approach is similar in other model organisms, and the differences will be reviewed at the end of this article.

## Strategies Used in the Candidate Gene Approach

### Selecting a Candidate Gene

The first critical step in conducting candidate gene studies is the choice of a suitable candidate gene that may plausibly play a relevant role in the process or disease under investigation. For example, when studying alcoholism, genes encoding enzymes that act in various pathways of alcohol metabolism, such as alcohol dehydrogenase (ADH) and aldehyde dehydrogenase (ALDH), are logical choices. Both enzymes are encoded by more than one gene (i.e., by gene families), and each of these genes exists in several variants, or alleles, allowing for its use in the candidate gene approach.

In alcoholism, as in other addictive disorders, the pathways through which brain chemicals (i.e., neurotransmitters) and other signaling molecules act may also play a role in the development and maintenance of addictive behaviors. For example, researchers using the fruit fly *Drosophila melanogaster* as an animal model of alcohol sensitivity found that a fly mutant called *cheapdate* exhibited enhanced vulnerability to alcohol (Moore et al. 1998). This mutant carries a specific allele of a gene important in cellular signaling pathways; consequently, genes involved in this and other signaling pathways would be reasonable choices for candidate genes influencing alcohol sensitivity and possibly alcoholism. (For more information on alcohol-related studies in *Drosophila*, see the article in this issue by Heberlein, pp. 185–188.) The selection of particular genes for further analysis as candidate genes could be facilitated if some of the potentially important genes were located in DNA regions that could be linked to alcoholism in genome screens.

### Choosing a DNA Polymorphism

Once investigators have selected a candidate gene, they must decide which polymorphism would be most useful for testing in an association study. To this end, they must identify existing gene variants and determine which of those variants result in proteins with altered functions that might influence the trait of interest.^1^ (For more information on the relationship between mutations in the DNA and variations in protein function, see the sidebar, p. 167.) In the case of ALDH, several well-known polymorphisms result in the substitution of certain protein building blocks (i.e., amino acids) and thus can lead to proteins with biologically relevant changes in function. In many cases, however, researchers may know a gene’s DNA sequence but may not have any information about functional variation in the gene.

**GENES and MUTATIONS**

DNA, the genetic material contained in each cell, encodes the information for all the proteins needed to create and maintain an organism. The information for each protein is contained within one gene. Genes represent only a small portion of a cell’s entire DNA (i.e., the genome), however, and stretches of DNA both between and within genes are not converted into proteins. Some of these “noncoding” DNA stretches (e.g., promoters) regulate the activity of the genes and determine which gene is turned “on” or “off ” in a given cell at a given time. This regulation is necessary, because not all cells need to generate all proteins at all times, and excessive or untimely protein production can lead to disease. For example, only blood cells need to produce the protein hemoglobin, which carries oxygen from the lungs to the tissues. Noncoding DNA stretches within genes are called introns. They are cut (i.e., spliced) out of an intermediary molecule called messenger RNA during the conversion of the genetic information in the DNA into a protein. This splicing process must be highly accurate in order to ensure that the resulting protein is functional.

DNA is a long, thread-like molecule whose building blocks—the nucleotides—consist of sugar molecules linked to organic bases. There are four such bases: adenine (A), guanine (G), cytosine (C), and thymine (T). The order, or sequence, in which the nucleotides are arranged specifies the order in which the building blocks of the resulting proteins (i.e., the amino acids) are combined. Because there are 20 amino acids but only 4 different nucleotides, a triplet of three nucleotides (i.e., a codon) represents one specific amino acid. However, the 4 nucleotides can be arranged into 64 different triplets, far more than the 20 codons needed to represent each amino acid. As a result, the genetic code is redundant, which means that more than one codon can represent a particular amino acid. For example, only the codon ATG represents the amino acid methionine; however, four different codons (GCA, GCC, GCG, and GCT) represent the amino acid alanine.

Both during the DNA duplication that occurs when cells divide and as the result of external factors (e.g., exposure to radiation or certain chemicals), changes in the nucleotide sequence (i.e., mutations) can occur. If these changes result in altered proteins that contribute to the development of a different phenotype, they represent polymorphisms that can be useful in candidate gene studies. Many mutations do not result in amino acid changes, however, and therefore do not alter the resulting protein or its function. For example, because of the redundant nature of the DNA code, some mutations result in codons that still specify the same amino acid. Thus, if a mutation occurred in the last nucleotide of the GCA triplet, all three possible new triplets (GCC, GCG, and GCT) would still encode the amino acid alanine. Nevertheless, these single nucleotide polymorphisms (SNPs) can be useful in linkage studies, as described in the main article.

Furthermore, many mutations occur in noncoding DNA regions and therefore do not result in protein variants that are associated with an altered phenotype or increased disease risk. Under two conditions, however, even mutations in noncoding regions might result in an altered phenotype and therefore be useful in candidate gene studies. First, mutations that occur in regulatory regions, such as promoters or intron splice sites, could alter gene activity and, consequently, the phenotype determined by that gene. Second, noncoding mutations that occur in an intron or outside a gene could be associated with an altered phenotype if they are positioned close to (i.e., typically within 200,000 nucleotides) a functional mutation and are therefore almost always inherited together with the functional mutation. This phenomenon is known as “linkage disequilibrium.” In all other cases, an observed association between a noncoding mutation and a disease may be a consequence of population stratification—that is, general differences between cases and controls if both subject groups are drawn from different underlying populations (e.g., ethnic groups or animal strains)—or a chance event.

—Jennifer M. Kwon and Alison M. Goate

Detecting genetic variants is a laborious process that often involves sequencing—that is, determining the sequence of DNA building blocks (i.e., nucleotides)—for the entire gene in both affected and unaffected individuals to look for consistent differences.^2^ Alternatively, researchers can employ screening procedures during which they isolate small gene sections from many individuals and compare their mobility in a gelatinous material. Differences in mobility in these analyses may indicate nucleotide variations (Malhotra and Goldman 1999).^3^ To confirm that a potential nucleotide variation exists and to determine its exact location in the genome, investigators then must conduct additional studies, typically on the direct sequencing of the DNA section in question. This information also allows researchers to determine whether the nucleotide variation is likely to have functional significance, either because it actually results in amino acid changes in the resulting protein or because it occurs in DNA regions controlling the gene’s activity. Finally, to be useful for candidate gene studies, the variant should occur with sufficient frequency to allow detection of differences between individuals with and without the trait under investigation.

Not all genes, however, have an easily identifiable common functional variant that can be exploited in association studies, and in many cases researchers have identified only changes in individual nucleotides (i.e., single nucleotide polymorphisms [SNPs]) that have no known functional significance. Nevertheless, SNPs can be potentially useful in narrowing a linkage region. In addition, they may show a statistically significant association with a disease susceptibility gene if they are located within or near that gene by virtue of linkage disequilibrium (see the sidebar for a description of this phenomenon).

SNPs can be of particular benefit in studies of complex disorders for which many potential candidate genes exist. For example, linkage mapping studies have suggested several genomic areas that may contain susceptibility genes for alcoholism. Each of these areas, however, is so large that it may contain dozens or hundreds of genes depending on the size and gene density of each region.^4^ Because it would be prohibitively difficult to sequence all these genes, publicly available SNP data are a great resource for candidate gene and association studies. For example, researchers recently analyzed several SNPs in the DNA region containing a candidate gene for Alzheimer’s disease (AD) and demonstrated that two SNPs closely flanking that gene indeed showed strong association with AD (Martin et al. 2000). (For more information on this candidate gene for AD, see the section “Examples of the Candidate Gene Approach in Humans.”)

### Testing the Candidate Gene

Once investigators have chosen a candidate gene and suitable polymorphism, they commonly test the role of this gene in a sample of randomly chosen subjects with the disease (i.e., cases) and without the disease (i.e., controls). Such subject groups are relatively easy to obtain, giving the candidate gene approach an important advantage over the linkage mapping approach, which requires the analysis of families with multiple affected members. Additional advantages of the case-control study design over linkage-based methods include the following (Malhotra and Goldman 1999):

- Researchers can more easily obtain large numbers of cases and control subjects.
- The effect of disease heterogeneity (i.e., that a disease may have multiple genetic causes despite a similar disease phenotype) is less problematic.
- Researchers do not need to make assumptions about the exact mode of disease transmission before conducting their analyses.

The major problem associated with the case-control design is that it may result in spurious associations if the controls are not appropriately matched to the cases with respect to ethnicity or other factors that influence an individual’s genetic composition.

## Examples of the Candidate Gene Approach In Humans

A widely cited example of the usefulness of the candidate gene approach involves AD, the most common cause of dementia in the elderly. AD typically is a late-onset disorder (i.e., the earliest symptoms occur after age 60) with a complex inheritance pattern. The disease often appears to occur sporadically, even when there is an underlying genetic predisposition. One of the pathologic hallmarks of AD is the presence of microscopic aggregates, or plaques, of a small protein-like molecule called β-amyloid peptide. These β-amyloid plaques also contain several other proteins, including one called apolipoprotein E (ApoE), whose gene (*APOE*) is located on chromosome 19. ApoE was implicated in the development of AD by findings that it binds tightly to β-amyloid in the fluid surrounding the brain and spinal cord (i.e., the cerebrospinal fluid) (Strittmatter et al. 1993). Furthermore, prior linkage data had indicated that a gene for late-onset AD was located on chromosome 19 (Pericak-Vance et al. 1991), in a region that included the *APOE* gene. Based on these findings, researchers conducted an association study comparing the frequency of three *APOE* alleles called E2, E3, and E4 in 30 cases and 91 unrelated controls (Strittmatter et al. 1993). The investigators found that whereas all alleles occurred in the controls, the APOE*E4 allele was greatly overrepresented in the AD cases, indicating that this allele is a major risk factor for the development of AD. This robust association between APOE*E4 and AD has been confirmed in many subsequent studies (for a review, see St. George-Hyslop 2000).

With respect to alcoholism, researchers have used the candidate gene approach to investigate the association between certain *ADH* and *ALDH* alleles and an altered risk of alcoholism. Studies have found that the enzyme encoded by an *ALDH* allele called ALDH2*2 degrades acet aldehyde more slowly than normal, resulting in the prolongation of certain unpleasant alcohol effects, such as facial flushing, racing of the heart (i.e., palpitations), and nausea. Not surprisingly, this allele appears to have a protective effect against alcoholism—that is, people carrying the allele are less likely to consume alcohol and to develop alcoholism (for a review, see Reich et al. 1999). The frequency of the ALDH2*2 allele is particularly high in some Asian populations, and carriers of this allele consume less alcohol and are much less likely to develop alcoholism than are people without the allele.

## The Candidate Gene Approach in Mouse Studies

Quantitative trait loci (QTLs) are DNA regions that may contain one or more genes related to the development of a certain quantitative trait. Mapping of QTLs in animal models of alcohol-related phenotypes has identified multiple genomic areas that potentially contain candidate genes for these phenotypes. (For more information on QTL mapping, see the article in this issue by Grisel, pp. 169–174.) The methods of identifying these candidate genes and any potential functional variants are essentially the same as those used in humans. Once functional variants are found, however, any positive association between a variant and the trait of interest must be interpreted with caution. For example, because of the way mouse strains are bred, mice who have a trait (analogous to human cases) and mice who do not have that trait (analogous to controls) may possess different alleles at a particular gene even if that gene is unrelated to the disease (or trait) under consideration. The gene that actually confers the risk for the disease or trait under investigation may be located near the gene showing the allelic polymorphism, but may be difficult to identify positively using association methods alone.

## Conclusion

A combination of linkage mapping and a candidate gene approach has been the most successful method of identifying disease genes to date. The candidate gene approach is useful for quickly determining the association of a genetic variant with a disorder and for identifying genes of modest effect. This approach has certain advantages over traditional linkage mapping or positional cloning approaches. The current methods for evaluating risk associated with candidate genes complement traditional linkage efforts in identifying susceptibility genes for alcoholism. As more SNPs are identified throughout the genome, some of those SNPs also will be located within candidate genes, thereby allowing researchers the use of the candidate gene approach on a genome-wide scale.

## Footnotes

1 Both the genes and the proteins they encode frequently are abbreviated with the same letters; however, the names of the genes are usually typed in italics and the names of the proteins in regular letters.

2 A typical gene can span 10,000 to 100,000 or more nucleotides of the human genome, of which approximately 2 to 5 percent (i.e., a few thousand nucleotides) consist of the coding sequence and the rest, intronic sequence.

3 Two such techniques are single strand conformation analysis (SSCP) and denaturing high performance liquid chromatography (DHPLC). For a discussion of other candidate gene variant selection techniques, see Malhotra and Goldman 1999; Collins et al. 1997.

4 Genes are not equally spaced throughout the genome, and some DNA regions may contain more genes than others. For example, although the human chromosomes 21 and 22 are of similar size (approximately 33.5 million nucleotides), their gene density differs substantially. Thus, there are on average 6 genes per million nucleotides on chromosome 21 (Hattori et al. 2000) but 16 genes per million nucleotides on chromosome 22 (Dunham et al. 1999).

## References

[b1-arcr-24-3-164] Collins FS, Guyer MS, Charkravarti A (1997). Variations on a theme: Cataloging human DNA sequence variation. Science.

[b2-arcr-24-3-164] Dunham I, Shimizu N, Roe BA (1999). The DNA sequence of human chromosome 22. Nature.

[b3-arcr-24-3-164] Malhotra AK, Goldman D (1999). Benefits and pitfalls encountered in psychiatric genetic association studies. Biological Psychiatry.

[b4-arcr-24-3-164] Martin ER, Gilbert JR, Lai EH (2000). Analysis of association at single nucleotide polymorphisms in the APOE region. Genomics.

[b5-arcr-24-3-164] Moore MS, DeZazzo J, Luk AY (1998). Ethanol intoxication in *Drosophila:* Genetic and pharmacological evidence for regulation by the cAMP signaling pathway. Cell.

[b6-arcr-24-3-164] Pericak-Vance MA, Bebout JL, Gaskell PC (1991). Linkage studies in familial Alzheimer disease: Evidence for chromosome 19 linkage. American Journal of Human Genetics.

[b7-arcr-24-3-164] Reich T, Hinrichs A, Culverhouse R, Bierut L (1999). Genetic studies of alcoholism and substance dependence. American Journal of Human Genetics.

[b8-arcr-24-3-164] Risch N, Merikangas K (1996). The future of genetic studies of complex human diseases. Science.

[b9-arcr-24-3-164] StGeorge-Hyslop PH (2000). Molecular genetics of Alzheimer’s disease. Biological Psychiatry.

[b10-arcr-24-3-164] Strittmatter WJ, Saunders AM, Schmechel D (1993). Apolipoprotein E: High avidity binding to β-amyloid and increased frequency of type 4 allele in late-onset familial Alzheimer disease. Proceedings of the National Academy of Sciences USA.

